# Hypermethylation-associated downregulation of microRNA-4456 in hypersexual disorder with putative influence on oxytocin signalling: A DNA methylation analysis of miRNA genes

**DOI:** 10.1080/15592294.2019.1656157

**Published:** 2019-09-22

**Authors:** Adrian E. Boström, Andreas Chatzittofis, Diana-Maria Ciuculete, John N. Flanagan, Regina Krattinger, Marcus Bandstein, Jessica Mwinyi, Gerd A. Kullak-Ublick, Katarina Görts Öberg, Stefan Arver, Helgi B. Schiöth, Jussi Jokinen

**Affiliations:** a Department of Neuroscience, Functional Pharmacology, Uppsala University, Uppsala, Sweden; b Department of Clinical Sciences/Psychiatry, Umeå University, Umeå, Sweden; c Andrology/Sexual Medicine Group (ANOVA), Department of Medicine, Karolinska Institutet, Stockholm, Sweden; d Department of Clinical Pharmacology and Toxicology, University Hospital Zurich, University of Zurich, Zürich, Switzerland; e Institute for Translational Medicine and Biotechnology, Sechenov First Moscow State Medical University, Moscow, Russia; f Department of Clinical Neuroscience/Psychiatry, Karolinska Institutet, Stockholm, Sweden

**Keywords:** Methylome-wide, DNA methylation, hypersexual disorder, MicroRNA, oxytocin signaling, oxytocin, psychiatry, hsa-miR-4456, microRNA-4456, MIR4456, epigenetic dysregulation, differential methylation, microRNA expression, gene target prediction, epigenetics

## Abstract

Hypersexual disorder (HD) was proposed as a diagnosis in the DSM-5 and the classification ‘Compulsive Sexual Behavior Disorder’ is now presented as an impulse-control disorder in ICD-11. HD incorporates several pathophysiological mechanisms; including impulsivity, compulsivity, sexual desire dysregulation and sexual addiction. No previous study investigated HD in a methylation analysis limited to microRNA (miRNA) associated CpG-sites. The genome wide methylation pattern was measured in whole blood from 60 subjects with HD and 33 healthy volunteers using the Illumina EPIC BeadChip. 8,852 miRNA associated CpG-sites were investigated in multiple linear regression analyses of methylation M-values to a binary independent variable of disease state (HD or healthy volunteer), adjusting for optimally determined covariates. Expression levels of candidate miRNAs were investigated in the same individuals for differential expression analysis. Candidate methylation loci were further studied for an association with alcohol dependence in an independent cohort of 107 subjects. Two CpG-sites were borderline significant in HD – cg18222192 (MIR708)(*p* < 10E-05,*p*_FDR_ = 5.81E-02) and cg01299774 (MIR4456)(p < 10E-06, *p*_FDR_ = 5.81E-02). MIR4456 was significantly lower expressed in HD in both univariate (p < 0.0001) and multivariate (p < 0.05) analyses. Cg01299774 methylation levels were inversely correlated with expression levels of MIR4456 (p < 0.01) and were also differentially methylated in alcohol dependence (p = 0.026). Gene target prediction and pathway analysis revealed that MIR4456 putatively targets genes preferentially expressed in brain and that are involved in major neuronal molecular mechanisms thought to be relevant for HD, e.g., the oxytocin signalling pathway. In summary, our study implicates a potential contribution of MIR4456 in the pathophysiology of HD by putatively influencing oxytocin signalling.

## Introduction

Hypersexual disorder (HD) was conceptualized as a non-paraphilic sexual behaviour disorder with impulsivity content and proposed as a diagnosis for inclusion in the DSM-5 [1]. The classification ‘Compulsive Sexual Behavior Disorder’ is now presented as an impulse-control disorder in ICD-11 [2]. HD incorporates different pathophysiological mechanisms including impulsivity, compulsivity, sexual desire dysregulation and sexual addiction. Today, little is known about the neurobiology behind this disorder. Focusing on neuroendocrine systems, hyperactive HPA axis assessed with the dexamethasone test [3]was found in men with hypersexual disorder and a recent study by Jokinen et al. examined methylation of hypothalamic-pituitary-adrenal (HPA) axis related genes in hypersexual disorder and discovered epigenetic changes in the CRH gene, an important integrator of neuroendocrine stress responses in the brain, with a key role in the addiction processes [4]. Drawing on the cumulative evidence from different scientific perspectives such as neuroimaging studies, neuropharmacological evidence, genetic as well as animal studies, Kühn et al. suggested that HD is associated with alterations in the dopaminergic system and specific regions of the brain, including the frontal lobe, amygdala, hippocampus, hypothalamus, septum and regions of the brain that process reward [5]. An increasing number of studies suggest a [5] significant role of epigenetic modifications, such as DNA methylation and histone acetylation, on sexual behaviour and human brain functioning [6–8]. Variations in DNA methylation modify gene expression by modulating the transcriptional expression or by recruiting specific proteins that remodel the chromatin state. Furthermore, methylation levels have been shown to influence the expression of fine-regulatory genes such as microRNAs (miRNAs) [9,10]. A further understanding of the interaction between DNA methylation, miRNA activity and HD may lead to new insights into the pathogenesis of this disorder that could contribute to novel treatment options to improve the clinical outcome of those affected.

Growing evidence suggests a role of DNA methylation in the pathophysiology of several psychiatric diseases, including major depressive disorder [11], bipolar disorder [12], psychosis [13] and post-traumatic stress disorder [14]. In support of this, shifts in epigenetic patterns have been shown to influence several aspects of CNS functions [15,16]. In the genome, DNA methylation refers to the addition of a methyl group to the 5ʹ-position of the cytosine pyrimidine ring. The extent of methylation can influence transcriptional activity by modulating the binding of transcription factors to the DNA, resulting in altered transcription of adjacent genes, or by altering chromatin states, affecting transcriptional activity of a larger cluster of genes. In animal models, epigenetic modifications have been shown to significantly affect sexual behaviour. A review by Matsuda et al. summarized literature that, among others, provided evidence that histone modifications and DNA methylation changes in the estrogen receptor α (ERα) can influence sociosexual behaviour [6]. Wang et al. showed that the formation of partner preference was improved by histone deacetylase-inhibitors in female prairie voles, an effect which was mediated by an epigenetically driven up-regulation of oxytocin receptor and vasopressin V1a receptor expression in the nucleus accumbens [7]. Zeh et al. further suggested that epigenetic variation could contribute to explaining post-copulatory sexual selection and linking sperm competitive ability to offspring fitness [8].

Recent studies have revealed altered miRNA expression profiles in the circulation and brain of patients with psychiatric disorders [17,18]. Drawing on this evidence, Issler et al. suggested that miRNAs targeting mRNAs expressed in brain could become novel treatment options in psychiatric disorders [19]. miRNAs are small non-coding RNA molecules of approximately 22 nucleotides in length. By inhibiting or degrading target mRNA molecules, miRNA are an efficient mode of post-transcriptional regulation which modulates the protein expression of more than 50% of the known protein-coding genes [20]. Certain miRNAs are capable of silencing or modulating expression levels of up to several hundred different genes [19]. Furthermore, these miRNAs are remarkably stable in body fluids [21] and exosomes export miRNAs outside of the cell into the circulation and can cross the blood-brain barrier [22]. As such, they represent a biological model whereby peripheral processes can potentially modulate the expression of mRNAs and proteins in brain, involved the pathogenesis of mental disorders and may act as biomarkers [23].

HD has not previously been investigated with regards to epigenomics and transcriptomics in a methylome-wide study approach. In this study, we perform a DNA methylation analysis of miRNA-associated CpG-sites in peripheral blood in order to examine if any DNA methylation patterns are associated with HD compared to healthy volunteers. As a second step, we measured the expressional profile of identified candidate miRNA to investigate whether they were differentially expressed in HD. As a third step, we investigated whether the methylation levels of candidate CpG-sites contributes to explaining the differential expression pattern of the associated miRNAs in HD. Associations of methylation and transcription were further validated in an independent cohort of 26 healthy volunteers. CpG sites that were differentially methylated by disease state (hypersexual disorder or healthy control) were further investigated for an association with alcohol dependence in an independent cohort of 107 subjects. An alcohol dependence cohort was chosen as validation data set mainly due to data availability reasons but also as this phenotype is believed to share common features with primarily the addictive component of HD.

## Methods

### Characterization of the discovery group

#### Ethics and patient consent

The study protocols were approved by the Regional Ethical Review Board in Stockholm (Dnrs: 2013/1335–31/2) and all participants gave their written informed consent to participate in the study. The study was performed in accordance with relevant guidelines and regulations.

#### HD patients

Details on the HD cohort have been previously published [3,4]. The study was performed at the Centre for Andrology and Sexual Medicine (CASM) at the Karolinska University Hospital, a multidisciplinary centre for diagnostics and treatment of patients with sexual dysfunctions. Seventy-four patients seeking medical and/or psychotherapeutic treatment were recruited in the study of neuroendocrine and epigenetic markers of HD. Inclusion criteria were a diagnosis of HD defined according to the DSM-5 proposed criteria for HD by Kafka et al. [1], age of 18 years or older and available contact information. Patients were excluded if they had any current psychotic illness, other psychiatric disease requiring immediate treatment, current alcohol or drug abuse and serious physical illness such as severe hepatic or renal disease. Subsequently, all patients were evaluated in a face to face interview by a trained psychiatrist and a psychologist using the Mini International Neuropsychiatric Interview (MINI) protocol [24] to establish psychiatric diagnoses.

#### Healthy volunteers

Healthy volunteers were recruited from the Karolinska Trial Alliance (KTA) database and as previously published [4]. Healthy volunteers were chosen to match HD patients by age and equal blood collection times in fall or spring to minimize seasonal variations. Subjects were included if they had no evidence of previous or current psychiatric illness, no serious physical illness, no first degree relative with bipolar disorder, completed suicide or schizophrenia; and no previous exposure to serious trauma. Volunteers screened positive for pedophilic disorder were excluded. In total, 39 male volunteers were included in the study.

#### Assessments

Both HD patients and healthy volunteers were assessed using the Mini-International Neuropsychiatric Interview (MINI 6.0) [24], the Hypersexual Disorder Screening Inventory (HDSI) (www.dsm5.org) [25], the Sexual Compulsivity Scale (SCS) [26], the Hypersexual Disorder: Current Assessment Scale (HD:CAS) [3], the Montgomery-Åsberg Depression Rating Scale Self rating (MADRS-S) [27] and the Childhood Trauma Questionnaire [28].

#### Blood sample collection, dexamethasone test and hormone level analysis

Blood samples were collected in the morning according to standard procedures from non-fasted participants. Analyses of plasma ACTH and Cortisol assays were performed directly after sampling at the laboratory of the Karolinska University Hospital using a chemiluminescence immunoassay. Low dose dexamethasone suppression tests (DST) were performed in all participants after the baseline plasma samples of ACTH and Cortisol were collected by administration of an oral dexamethasone (0.5 mg) at 23.00 h the same day. The following day, post DST blood samples were collected at approximately 08.00 h. A plasma Cortisol level of 138 nmol/l (= 5 g/dl) or higher in the morning sample after dexamethasone administration classified as non-suppressed.

### Methylation profiling

Genomic DNA was extracted from whole blood of 110 samples using the phenol-chloroform method [29]. Subsequently, the EZ DNA Methylation – GoldTM kit (ZymoResearch, USA) was used for bisulphite conversion. Bisulphite converted DNA was thereafter hybridized to the Illumina Infinium Methylation EPIC BeadChip, measuring the methylation state of over 850 K CpGsites. The arrays were imaged and analysed using the Illumina iScan system (Illumina, San Diego, CA, USA) in which the per cent methylation state of each CpG-site was quantified.

#### Data processing

Preprocessing of the methylation data was performed by background correction, adjustment of probe type differences, removal of batch effects and probe exclusion. Subsequently, the global DNA methylation pattern was adjusted for white blood cell type heterogeneity. Principal component analysis (PCA) was used to identify sample outliers in the methylation data. Methylation preprocessing steps were performed using the minfi [30], watermelon [31], sva [32], ChAMP [33] of the Bioconductor project and the FactoMineR [34] package of the CRAN project operable in R, version 3.3.0. For all other statistical analysis please see Supplementary material concerning background correction, adjustment of type I and type II probes, removal of batch effects and probe exclusion and correction for white blood cell type heterogeneity and criteria of sample exclusion.

#### CpG-site annotation and selection of miRNA-associated probes

Ninety per cent of the probes on the Illumina 450K Methylation Beadchip are also present on the Illumina EPIC BeadChip array [35]. The annotation for the EPIC Beadchip provided by Illumina does not provide information about the information about the distance to the TSS. In addition, for some CpG sites the EPIC annotation may refer to more than one associated gene. We therefore used the expanded annotation produced by Price et al., originally designed for the 450K array, to define, for each CpG-site, the distance to the closest transcriptional start site (TSS) and the associated gene [36]. As such, only CpG-sites present on the Illumina 450 K methylation beadchip were considered for further analysis. After the preprocessing steps outlined above, all CpG-sites annotated to any known miRNA were included in the study, resulting in 8,852 miRNA-associated CpG-sites investigated in the subsequent analysis.

### Mirna profiling

#### RNA extraction

800 ul of thawed EDTA blood samples stored at 80° C were centrifuged for 10 min at 1900 g and subsequently for 10 min at 16’000 g (4°C). To extract RNA from 200 µl cell-debris-free supernatant the miRNAeasy Serum/Plasma Kit (Qiagen, Hilden, Germany) was used according to the manufacturer’s instructions.

#### Reverse transcription and quantitative real-time PCR

To measure miRNA expression, 10 ng of extracted RNA was reverse transcribed into cDNA using a TaqMan® miRNA Reverse Transcription Kit (Applied Biosystems, Waltham, MA, USA) and specific stem-loop reverse transcription primers (TaqMan® MicroRNA Assays for MIR708-5p and MIR4456; Life Technologies, Carlsbad, CA, USA). The following running conditions were applied: 16°C 30 min, 42°C 30 min, 85°C 5 min. RT-PCR was performed using 0.67 µl cDNA and 9.3 µl RT-PCR Universal Fast Master Mix (Applied Biosystems) including the respective miRNA-specific primers. The following RT-PCR running conditions were applied: 95°C 20 s (95°C 1 s, 60°C 20 s) x 40; heating steps 1.9°C/s; cooling steps 1.6°C/s. U6snRNA was used as an internal control. Analysis was performed using the ViiA™ 7 Real-Time PCR System (Applied Biosystems). All measurements were performed in triplicate.

#### Sample exclusion considerations

Of the 93 samples included in the epigenome-wide analysis, two samples did not have enough blood to perform the RNA extraction. An additional two samples were switched for two samples that were not included in the epigenome-wide analysis. Furthermore, two additional samples were excluded as these due to methodological reasons were treated with an adjusted centrifugation protocol, resulting in six samples being excluded and a total sample size of 87 individuals included in the subsequent analysis of expression levels. In the case of the MIR708 expression levels, 13 of the remaining subjects exhibited cycle numbers greater than 35 in two values out of the measured triplicate and were thus excluded from the analysis as they were considered unreliable. As a final step, we performed boxplot diagrams of the expression levels and excluded four outliers in the MIR4456 data and eight outliers in the case of MIR708. After the exclusion steps, 83 subjects for MIR4456 (55 HD and 28 healthy volunteers) and 66 subjects for MIR708 (45 HD and 21 healthy volunteers) were included in the subsequent analysis of expression levels.

### Characterization of the validation data set

Data is openly available (E-GEOD-72680) and were originally published by Kilaru et al. [37]. The study included only African American patients with the goal of identifying associations between peripheral blood DNA methylation and psychiatric symptoms. Blood was collected in EDTA vacuum tubes prior to extraction. DNA methylation was assessed in whole blood using the Illumina 450 K methylation beadchip from participants of the Grady Trauma Project. For the purpose of this study, apart from the DNA methylation data, we made use of the following phenotypes: age, gender, BMI, the Kreek-McHugh-Schluger-Kellogg (KMSK) scale [38] measured for alcohol, and the occurrence of any under treatment depressive disorder, anxiety disorder, bipolar disorder or post-traumatic stress disorder. We also made use of the supplied relative proportions of white blood cell coefficients: B-cells, CD4-T cells, CD8 T-cells, granulocytes, monocytes and NK-cells. Please see https://www.ebi.ac.uk/arrayexpress/experiments/E-GEOD-72680/for a list of all available phenotypes.

### Statistical analysis

All statistical analyses were performed using R statistics, version 3.3.0.

#### Data analysis

As a proxy variable for ethnicity, subjects were stratified as being of ‘Scandinavian descent’ if born in a Scandinavian country (e.g., Denmark, Finland, Iceland, Norway or Sweden) and with biological parents born in the same country. Skewness and kurtosis of the distribution of continuous variables were evaluated with the Shapiro–Wilks test. Baseline cortisol levels in both HD patients and healthy volunteers and HbA1C (mml/mol) in controls were normally distributed, whereas the other clinical variables were not. Thus, the t-test was used to investigate group differences in baseline cortisol levels and HbA1C (mml/mol) and the Kruskal–Wallis’ test was used to investigate group differences in the other continuous variables between HD patients and healthy controls in an unadjusted manner. Chi-squared tests were used to detect differences in categorical variables, e.g., gender, depression and DST non-suppression status.

#### Adjusting for potential confounders

We considered initially the following variables as potential confounders in association analysis between DNA methylation and HD, i.e., depression, DST non-suppression status, CTQ Total, ACTH levels after the dexamethasone suppression test (DST cortisol, DST ACTH), plasma levels of testosterone, HBA1C, TSH, TNF-alpha and IL-6. To avoid overfitting by including too many covariates, we first investigated each individual covariate against the phenotype of interest in binary logistic regression models using the ‘glm’ function in R. Covariates were incrementally and independently selected. Only covariates with near significant between-group differences (p < 0.10) were considered in the analysis. Using the computed analysis of variance, we tested whether the addition of a particular covariate resulted in a better fit to the model and only included variables with a p-value < 0.05. Age was used as a base covariate for the regressions. The best linear model for hypersexuality included depression (p = 0.0113), DST outcome (p = 0.003517), CTQ total (p = 0.0273) and TNF-alpha (p = 0.01437). Three individuals lacked data for TNF-alpha and two individuals lacked data for CTQ total – both were excluded from the analysis resulting in a total sample size of 88 individuals used in the subsequent analysis.

#### DNA methylation association study

The association analyses between DNA methylation and hypersexuality were tested with linear models using the ‘limma’ package for R, applying an empirical Bayes method based on a moderated t-statistic [39,40]. These linear regressions using limma have been previously considered fitting for large-scale methylation studies [39,40]. For statistical analysis, we transformed the beta values to M-values, which have been shown to be statistically more robust [41]. We assumed a linear model where the M values of each CpG-site were used as a quantitative dependent trait and the phenotype of interest, e.g., hypersexuality, were used as covariates together with the other covariates determined to fit to the model. Standard errors were calculated manually by multiplying the square root of the posterior values for sigma^2 with the standard deviation. All analyses were accounted for multiple testing using the false discovery rate (FDR) method [42]. As a next step, the R/Bioconductor package *BACON* [43] was implemented to adjust the regression data for estimated bias and inflation. As a third step, as each gene transcript may be associated with several CpG-sites, we analysed the results of the regression analyses to identify gene transcripts with an abundance of differentially methylated CpG-sites using binomial tests. P-value thresholds were set to 0.05 to stratify probes according to significant and non-significant methylation changes. Subsequently, binomial tests were performed in R using the function ‘binom.test’, contrasting for each gene the number of nominally significant CpG-sites to the total number of probes annotated to each gene transcript not taking the direction of the methylation change into account. Binomial test p-values were adjusted for multiple testing using the FDR-method [42]. Gene transcripts with an FDR-adjusted binomial test p-value < 0.05 were considered significant.

#### Post-hoc analysis of main DNA methylation study

Recent studies have indicated that the ComBat function used for adjustment of batch effects in DNA methylation data can overcorrect data [44], potentially inflating resulting statistics. To ensure this was not a source of bias in our main analysis, we excluded ComBat from the analysis pipeline and performed the same regression analyses as described in section 2.5.3. Furthermore, we tested whether the exclusion of female samples would impact significantly on the key results. In a third analysis, all previously excluded samples were included in the analysis to investigate the impact of sample exclusion on downstream results. In a fourth analysis, we included the above described proxy variable for ethnicity as a co-variate to see if this would impact regression results. Lastly, in a fifth post-hoc analysis, additional microRNA annotated CpG-sites on the EPIC array were added to the analysis. For this final analysis, we made use of the Illumina Manifest File for the EPIC array. 19627 CpG-sites annotated to X and Y chromosomes were removed. Thereafter, an additional 129151 probes with known SNP sites with a minor allele frequency exceeding 0.05 were excluded from the analysis. A list of all known microRNA transcripts for Homo Sapiens was obtained from miRBase [45]. Remaining CpG-sites annotated to any known human microRNA were included in the analysis, resulting in 1465 additional microRNA-associated CpG-sites. Two hundred and sixty-eight of these were excluded during the preprocessing of the methylation data, resulting in 1197 additional CpG-sites added to the DNA methylation analysis. In total, 10049 (8852 + 1197) CpG-sites were studied. For all post hoc analyses presented above, the same analysis pipeline was followed as that described previously.

#### Validation cohort analysis

Of the 392 subjects included in the study, we first excluded 158 subjects lacking KMSK data. Following this step, we further excluded 127 subjects with a BMI exceeding 30 kg/m^2^, resulting in 107 individuals investigated in the subsequent analysis. Next, we stratified subjects into ‘alcohol dependent’ or ‘controls’ based on their score for alcohol on the KMSK. An arbitrary cut-off of 8 was used in order to achieve sufficient power for the subsequent analysis. Tang et al. demonstrated that this cut-off resulted in a sensitivity for alcohol dependence of 98.6%, a specificity of 58.2%, a positive predictive value of 0.538 and a negative predictive value of 0.988 [38]. To exclude any potential confound from white blood cell type heterogeneity, which has been shown to impact the DNA methylation pattern [40], we compared the relative proportions of B-cells, CD4 T-cells, CD8 T-cells, granulocytes and monocytes between subjects stratified as alcohol dependent and controls using unpaired t-tests. We then considered initially the following variables as potential confounders on the association analysis between DNA methylation and alcohol dependence, i.e., age, gender, BMI, and the occurrence of any under treatment depressive disorder, anxiety disorder, bipolar disorder or post-traumatic stress disorder. These parameters were compared between the groups in using unpaired t-tests and chi-squared tests. We thereafter performed binomial logistic regression models, contrasting disease status (alcohol dependence or control) to candidate CpG-site methylation levels and adjusting for the co-variates that exhibited significant between-group differences.

### Investigation of the expressional profile of candidate miRNAs

TaqMan miRNA Reverse Transcription Kit (Applied Biosystems, Waltham, MA, USA) was used to measure expression levels miRNAs that were associated with differentially methylated CpG-sites identified in the epigenome-wide analysis. Skewness and kurtosis of the distribution of the expression pattern were evaluated with the Shapiro–Wilks test. The measured miRNAs were not normally distributed. The Kruskal–Wallis’ test was, thus, used to investigate group differences in the expressional profile between HD patients and healthy controls. In addition, we performed binomial logistic regressions by contrasting expression levels between hypersexual patients and healthy volunteers and adjusting for both continuous variables, i.e. CTQ total and TNF alpha, as well as the categorical co-variates, i.e., depression and DST non-suppression status. Candidate CpG-sites were further investigated with regard to their association with transcriptional expression of the respective miRNA. We assumed a linear model, where the expression levels of each miRNA (dependent variable) were correlated to M values of the associated CpG-site (independent variable) intraindividually, adjusting for a categorical variable of disease state (HD or healthy volunteer) and an interaction term between methylation and disease state. As a third step, we also correlated miRNA expression levels with M values of the associated CpG-site in the combined Expression data set, adjusting for a categorical experimental variable.

#### Target gene prediction and pathway analysis

We used the online webtool ComIR to identify putative mRNAs putatively targeted by miRNAs in focus, a miRNA target prediction tool integrating the results from several distinguished target prediction algorithms such as TargetScan, Miranda, PITA and mirSVR [46]. Genes were considered as putative targets if the calculated equal abundance score exceeded 0.9, as performed by Maffioletti et al. [47]. Subsequently, genes identified as putative miRNA targets were investigated by overrepresentation analysis of tissue-specific gene expression profiling and KEGG-defined pathways, using the online web tools ‘DAVID Functional Annotation Bioinformatics Microarray Analysis’ [48] and ‘ConsensusPathDB-human’ [49]. For the overrepresentation analysis in KEGG-defined pathways, we specified a ‘minimum overlap with input list’ of 15 and a stringent p-value cut-off of 0.0001. To investigate if there were overlapping genes in the identified KEGG-defined pathways, we performed a heatmap analysis of the number of overlapping MIR4456 associated genes in each pathway using the R package ‘gplots’ [50].

#### Evolutionary conservation of candidate microRNA:s

BLASTn searches [51] were performed through the Ensembl (word size = 4 with all other parameters default) [52] and NCBI (word size = 16 with all other parameters default) (v. 2.5.0) webservers. The relevant hits were retained considering e-value, sequence identity and appropriate sequence coverage, i.e., if the seed region of the mature miRNA was covered. The 10 species with genomic assemblies include: Homo sapiens (GRCh38); Pan troglodytes (GSAC 2.1.4); Pan paniscus (panPan1); Gorilla gorilla gorilla (WTSI gorGor3.1); Pongo pygmaeus abelii (PPYG2); Chlorocebus sabaeus (VGC ChlSabeus1.1); Macaca mulatta (Mmul_8.0.1); Papio anubis (PapAnu_2.0); Saimiri boliviensis (saiBol1.1); and Callithrix jacchus (C_jacchus3.2.1). The sequence regions of interest were downloaded and then aligned using the Mafft webserver (v 7) [53] with the L-INS-i method with default settings. Visualization with nucleotide colouring scheme and editing of the alignment was performed in Jalview [54] with further annotation performed in Adobe Illustrator. The species phylogenetic tree was created using phyloT [55] and is based on NCBI taxonomy. The cladogram represents the evolutionary relationship among the investigated organisms; the branch lengths are not relative to evolutionary distances. The gene synteny was gleaned through the NCBI map view web portal [56] and through the UCSC genome browser [57].

## Results

### Study sample characteristics

In the Discovery cohort, comprising 60 patients diagnosed with hypersexual disorder (HD) and 33 healthy volunteers, we initially aimed to identify miRNA genes in proximity of CpG-sites, in which modifications of the epigenetic profile are associated with HD. Patients with HD had significantly more depression (p < 0.05), higher scores on the CTQ assessment (p < 0.001), higher levels of plasma ACTH after the DST (p < 0.01) and TNF-alpha (p < 0.0001), but lower levels of plasma IL-6 (p < 0.001). In addition, the HD patient group tended to have more DST non-suppressors as compared to control group (p = 0.058). There were no significant differences between groups in age, plasma testosterone levels, TSH/T4-quota, HbA1C, baseline cortisol, DST cortisol and baseline ACTH (Table 1)

**Table 1.** Clinical characteristics of patients with hypersexual disorder and healthy volunteers.

	Patients	Healthy volunteers	Statistics (t-test, Kruskall-Wallis, Chisq.test), p value
N	60	33	
Age (years)	39.4 (11.9)	37.4 (11.3)	*ns*
Men:Women, (n (%))	54 (90.0): 6 (10.0)	33 (100.0): 0 (0.0)	*ns*
Scandinavian descent (n(%))*	43 (71.7)	23 (69.7)	*ns*
Diagnosis depression (n(%))	9 (0.15)	0 (0.0)	**4.83E-02**
DST non-suppressors (n(%))	16 (26.7)	3 (9.1)	5.78E-02
CTQ Total	40.42 (11.89)	32.85 (9.39)	**2.22E-04**
TSH (mE/L)/T4 (nmol/L)	0.019 (0.0098)	0.027 (0.033)	*ns*
HBA1C (mml/mol)	32.97 (5.70)	32.82 (3.96)	*ns*
Cortisol (nmol/L)	467.85 (132.33)	474.49 (148.32)	*ns*
DST Cortisol (nmol/L)	100.4 (101.3)	62.54 (48.54)	*ns*
ACTH (pmol/L)	6.36 (3.05)	5.82 (2.99)	*ns*
DST ACTH (pmol/L)	2.11 (1.58)	1.26 (0.90)	**8.10E-03**
Testosteron (nmol/L)	14.02 (5.86)	14.24 (4.37)	*ns*
TNF-alpha (ng/L)	7.26 (1.83)	5.84 (2.41)	**4.39E-06**

Values are shown as mean (SD) unless otherwise specified. P-values were calculated by means of unpaired t-tests, Kruskall-Wallis’ test or chi-squared tests, contrasting values for patients with hypersexuality disorder and healthy volunteers. A one-tailed p-value <0.05 was considered significant.

*As a proxy for ethnicity, subjects born in a Scandinavian country (e.g., Denmark, Finland, Iceland, Norway or Sweden) and with biological parents born in the same country, were stratified as being of ‘Scandinavian descent’.

Abbreviations: CTQ, childhood trauma questionnaire; DST, dexamethasone suppression test; ACTH, ACTH levels after the dexamethasone suppression test; DST Cortisol, cortisol levels after the dexamethasone suppression test; DST non-suppressors, non-suppression status defined as DST cortisol levels >138 nmol/l

The Validation cohort comprised 107 subjects, of which 24 were stratified as alcohol dependent and 83 as controls based on the results on the Kreek-McHugh-Schluger-Kellogg (KMSK) score for alcohol. There were no between-group differences in cell-type proportions of B-cells, CD4 T-cells, CD8 T-cells, granulocytes, monocytes or NK-cells as measured by unpaired t-tests (data not shown). Controls had a significantly higher ratio of females (p < 0.01) and higher occurrence of subjects under treatment for anxiety disorders (p < 0.05). There were no between-group differences in age, BMI or in the occurrence of any under treatment depressive disorder, bipolar disorder or post-traumatic stress disorder (**Supplementary Table 1**).

### Two CpG-sites linked to MIR708 and MIR4456 are differentially methylated in subjects with hypersexual disorder

On the association analysis between DNA methylation of miRNA coupled CpG-sites and HD, we performed multiple linear regression models of methylation M-values to HD and adjusted for depression, DST non-suppression status, CTQ total score and plasma levels of TNF-alpha. The initial analysis of the 8,852 individual CpG-sites tested, yielded two CpG loci that were identified and associated with the miRNAs MIR708 and MIR4456, respectively, which were differentially methylated after correction for multiple testing using the FDR-method (p_FDR_<0.05)(Table 2**)(Supplementary Figure 1**). Cg18222192 (MIR708) was significantly hypomethylated in subjects with HD, whereas cg01299774 (MIR4456) exhibited a hypermethylation in the patient group. A Q-Q plot of the moderated t-statistic further suggested some inflation in the methylation analysis (**Supplementary Figure 2**.). Using R/Bioconductor package *BACON* yielded estimated bias of −0.082 and an estimated inflation of 1.1. Adjusting for this potential hidden confound yielded considerably less nominally significant CpG-sites (77 compared to 695) and resulted in the CpG sites above reaching FDR_adjusted p-values of 5.81 E-02. The BACON adjusted values yielded an estimated inflation of 1.0 and a minimal bias of 0.027. As a next step, we tested whether any individual miRNA had a statistically significant abundance of differentially methylated CpG-sites using binomial tests and a p-value threshold of 0.05. No individual miRNA had a statistically significant abundance of differentially methylated CpG-sites after adjustments were made for multiple testing. The five most significant miRNA:s were mir-133b, MIR4456, MIR124-1, MIR100HG and MIRLET7BHG. With 8 out of 33 CpG-sites differentially methylated in HD (p-value threshold of <0.05), MIR4456 was nominally significant in this respect (p < 0.001)(**Supplementary Table 2**).

**Table 2.** Hypersexuality associated methylation changes in miRNA-associated CpG sites.

			% DNA Methylation (SD)								
Gene	Transcript	Illumina ID	Patients	Healthy Volunteers	logFC	ES	SE	*p*	*p* (FDR)	*p_BC_*	*p_BC_* (FDR)
**MIR708**	**NR_030598**	**cg18222192**	**50.1 (3.0)**	**51.5 (3.7)**	**−0.11**	−0.11	2.35E-02	**7.04E-06**	**4.14E-02**	**1.31E-05**	5.81E-02
**MIR4456**	**NR_039661**	**cg01299774**	**51.3 (3.4)**	**48.7 (4.2)**	**0.12**	0.12	2.64E-02	**9.36E-06**	**4.14E-02**	**8.97E-06**	5.81E-02
MIR3141	NR_036094	cg05945665	65.3 (1.6)	66.1 (2.3)	−0.09	0.08	2.01E-02	**3.75E-05**	8.13E-02	**6.72E-05**	1.17E-01
Mir_584	.	cg27571590	73.3 (1.2)	74.0 (1.8)	−0.06	0.06	1.53E-02	**4.78E-05**	8.13E-02	**3.66E-04**	2.70E-01
MIR4710	NR_039860	cg16761754	38.5 (9.1)	34.0 (12.7)	0.35	−0.09	1.97E-02	**4.88E-05**	8.13E-02	**7.73E-05**	1.17E-01
MIR4278	NR_036242	cg08313382	58.5 (1.8)	57.6 (2.7)	0.08	0.09	2.27E-02	**5.99E-05**	8.13E-02	**1.19E-04**	1.17E-01
MIR345	NR_029906	cg02540736	53.2 (1.4)	54.1 (2.5)	−0.06	−0.07	2.08E-02	**6.43E-05**	8.13E-02	**1.42E-03**	4.69E-01
MIR4281	NR_036239	cg20960405	23.6 (1.9)	23.0 (2.8)	0.09	0.09	2.30E-02	**9.29E-05**	8.28E-02	**1.07E-04**	1.17E-01
Mir_1302	.	cg08241360	59.0 (4.1)	57.3 (6.5)	0.12	−0.03	1.11E-02	**9.92E-05**	8.28E-02	**1.06E-02**	6.71E-01
MIR4441	NR_039643	cg20871441	73.5 (1.0)	73.8 (1.0)	−0.03	0.04	1.19E-02	**1.01E-04**	8.28E-02	**1.85E-03**	4.69E-01

Analysis by multiple linear regression models of methylation M-values to a binary outcome variable of Hypersexuality (Y/N), adjusting for Depression (Y/N), dexamethasone suppresser (Y/N), CTQ total score and TNF-alpha (ng/L). 8,852 miRNA associated CpG-sites were analysed. Standard errors were calculated manually by multiplying the square root of the posterior values for sigma^2 with the standard deviation. P-values were corrected for multiple testing using the false discovery rate (FDR)-method. Correction for inflation and estimated bias was made using the package ‘Bacon’ for R statistics.

Abbreviations: logFC, log fold change of M-values; % DNA Methylation (SD), mean beta value percent DNA methylation (standard deviation); ES, effect size; SE, standard error; p, p-value; p (FDR), FDR-adjusted p-value; p_BC_, p-values after bacon adjustment for inflation and bias; p_BC_ (FDR), FDR-adjusted p-values after bacon adjustment for inflation and bias.

#### Post-hoc analyses suggest minimal bias from batch adjustment, inclusion of female samples, exclusion of outlier samples and ethnicity

Excluding ComBat from the analysis pipeline resulted in an estimated inflation of 1.3 and a total of 32 FDR-adjusted significant CpG-sites (pFDR < 0.05) prior to ‘bacon’-adjustment (compared to two FDR-adjusted significant CpG-sites when implementing ComBat in the analysis pipeline). This represents considerably more inflation than the ComBat adjusted estimated inflation of 1.1. Likewise, the exclusion of female participants resulted in MIR4456 associated CpG-site cg01299774 being the most significant probe prior to adjustment for bias/inflation (p = 2.053995e-05, pFDR = 0.1094308) albeit not significant after FDR-adjustments. Similar results resulted were obtained when including outlier samples previously excluded. Furthermore, including a categorical variable of ‘Scandinavian descent’ (yes or no) in the methylation analysis did not significantly alter the results for the MIR4456 associated CpG-site in focus, cg01299774 (p = 8.74E-06, pFDR = 0.049), measured before inflation/bias adjustment. For this analysis, estimated inflation was 1.1 and estimated bias −0.085. After adjustment for inflation/bias and FDR-adjustment, p-value for cg01299774 was 0.098 and the magnitude and direction of the methylation change was the same. Lastly, the inclusion of additional microRNA-associated CpG-sites annotated to the EPIC array resulted in MIR708 associated CpG-site cg18222182 (p = 7.23E-06, pFDR = 0.046) and MIR4456 associated CpG-site cg01299774 (p = 9.28E-06, pFDR = 0.046) to be significant after FDR adjustments but prior to inflation/bias adjustment. Inflation was 1.1 and bias −0.086. No individual CpG-site remained significant after inflation, bias and FDR-adjustment. The full results of all the above-presented post-hoc analyses are presented in Supplementary Materials.

### MIR4456 is lower expressed in peripheral blood of subjects diagnosed with hypersexual disorder

We measured the expression levels of MIR708 and MIR4456 in blood and compared them between controls and subjects diagnosed with HD. Due to hemolysis of the erythrocytes, it was not possible to separate the plasma from the corpuscular part of the blood. As such, the miRNA expression levels are measured on miRNA from plasma and blood cells. This, however, was the case for all our samples and should therefore not be a source of any occurring between-group bias.

**Figure 1.**
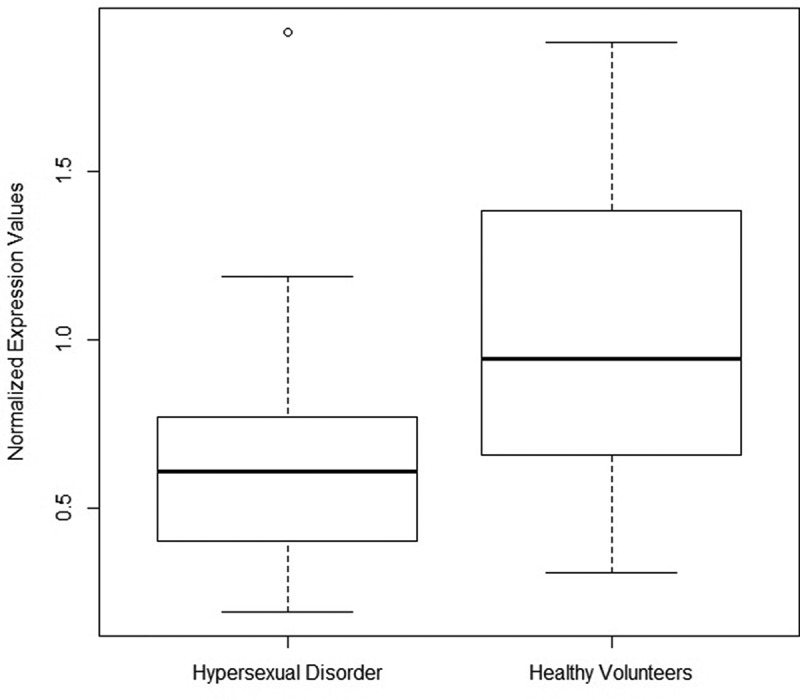
Boxplot diagram of MIR4456 normalized expression values in HD and healthy volunteers.

As neither of the two miRNAs were normally distributed, we used the Kruskal–Wallis’ test to investigate univariate between-group differences in expression levels. MIR4456 (p < 0.001) was significantly less expressed in subjects with HD, but there were no group differences in the expression of MIR708 (**Supplementary Table 3**)(Figure 1). In the following step, we performed binomial logistic regression analyses, contrasting miRNA expression levels by disease state (HD or healthy volunteer) and adjusting for depression, DST non-suppression status, CTQ total and TNF-alpha as co-variates. In this analysis, MIR4456 (p < 0.05) remained significantly lower expressed **(Supplementary Table 4**).

**Table 3.** Methylation/transcription correlations in HD patients and healthy volunteers of candidate CpG sites and MIR708 and MIR4456.

	MIR708				MIR4456			
	Coef.	Std. Error	t value	p.val	Coef.	Std. Error	t value	p.val
Intercept	**0.64**	**0.08**	**7.62**	**1.82E-10**	**0.79**	**0.06**	**13.5**	**< 2e-16**
cg18222192^a^/cg01299774^b^	0.63	0.40	1.56	0.13	**−0.72**	**0.25**	**−2.84**	**5.76E-03**
Disease state^c^	−0.13	0.10	−1.40	0.17	**−0.29**	**0.07**	**−3.94**	**1.78E-04**
Interaction term^d^	−0.06	0.47	−0.12	0.90	**0.90**	**0.33**	**2.75**	**7.37E-03**

Multiple linear regressions whereby the expression of each miRNA was correlated to M values of the CpG site intraindividually, adjusting for a categorical variable of disease state and an interaction term between methylation and disease state. miRNA expression levels were treated as dependent variable and DNA methylation as independent variable.

^a^cg18222192 in the case of MIR708

^b^cg01299774 in the case of MIR4456

^c^A categorical variable of disease state (hypersexuality disorder or healthy volunteer)

^d^Interaction term between CpG site methylation and a categorical variable of disease state

Abbreviations: coef., regression coefficient; p, p-value

**Table 4.** Gene set overrepresentation analysis of MIR4456 putative binding targets – KEGG defined pathways.

KEGG Pathway (Homo sapiens)	Set size	Count	%	p-value	q-value	source
Oxytocin signaling pathway	159	24	15.2%	7.29E-06	1.82E-04	KEGG
cAMP signaling pathway	200	26	13.0%	5.12E-05	4.14E-04	KEGG
Glutamatergic synapse	114	18	15.8%	5.92E-05	4.14E-04	KEGG
Circadian entrainment	96	16	16.7%	7.76E-05	4.14E-04	KEGG
Adrenergic signaling in cardiomyocytes	149	21	14.1%	8.28E-05	4.14E-04	KEGG

The online web tool ComIR’ was used to computationally predict putative gene targets of MIR4456. Using the online web tool ‘ConsensusPathDB-human’, 1142 identified genes were investigated to see if there was a statistically significant abundance of genes involved in specific KEGG-defined pathways. We specified a ‘minimum overlap with input list’ of 15 genes and a stringent p-value cut-off of 0.0001.

Abbreviations: Count, number of candidate genes in a particular pathway; %, percentage of candidate genes in a particular pathway

### Expression levels of MIR4456 are significantly associated with the methylation state of cg01299774 and the direction of the association is dependent on disease state

To evaluate to what extent the expression levels of MIR708 and MIR4456 are associated with the methylation state of the candidate CpG-sites, we performed multiple linear regressions whereby each miRNA was correlated to M-values of the associated CpG-site, adjusting for disease state (hypersexual disorder or healthy volunteer) and an interaction term between methylation status and disease state. Cg01299774 was inversely correlated (p < 0.01) with expression levels of MIR4456. Specifically, the interaction term composed of disease state and CpG-site methylation levels had a positive coefficient (p < 0.01), indicating disease state-dependent effects on the direction of the association between methylation status and expression level. Cg18222192 was not associated with MIR708 expression levels (Table 3).

### A significant overrepresentation of MIR4456 putative gene targets are preferentially expressed in brain and involved in major neuronal molecular mechanisms thought to be relevant to HD

Using the ComIR analysis software [46], we retrieved the predicted mRNA targets for MIR4456. 1,142 mRNAs were identified as putative targets for MIR4456 and were subsequently investigated by overrepresentation analysis of tissue-specific gene expression and KEGG-defined pathways. There was a statistically significant overrepresentation of genes expressed in brain (569 genes, q-value<0.00001), amygdala (59 genes, q < 0.01), epithelium (201 genes, q < 0.01), teratocarcinoma (50 genes, q < 0.05) and hippocampus (44 genes, q < 0.05)(**Supplementary Table 5**.). Gene set overrepresentation analysis further revealed an overrepresentation of genes associated with five KEGG-defined pathways, including oxytocin signalling pathway (24 genes, q < 0.01), cAMP signalling pathway (26 genes, q < 0.01), glutamatergic synapse (18 genes, q < 0.01), circadian entrainment (16 genes, q < 0.01) and adrenergic signaling in cardiomyocytes (21 genes, q < 0.01)(Table 4). To investigate the occurrence of overlapping genes between these pathways, we performed a heatmap based on the number of overlapping MIR4456 associated genes in each pathway. There appeared to be three main clusters of pathways (**Supplementary Figure 3**.). Importantly, the oxytocin signalling pathway, which included 24 MIR4456 associated genes, had a maximum overlap of only 13 genes with the other pathways.

### MIR4456 appears to be evolutionary conserved throughout primates

As miRNA4456 is identified in miRBase only in *Homo sapiens*, we performed a BLASTn [58] search using the 43-nucleotide stem-loop region to investigate the evolutionary conservation of MIR4646. This resulted in 10 species with relevant hits (**Supplementary Figure 4**.) with particular emphasis on the conservation in the seed region. All of the sequences are predicted to form a hairpin secondary structure. The last common ancestor of these species appears to be at the advent of primates. With the conservation in the multiple sequence alignment and the predicted hairpin secondary structures, this might suggest that this region is conserved from throughout primates.

### MIR4456 associated CpG-site cg01299774 is hypermethylated in alcohol dependence in the validation cohort

Candidate CpG loci that were differentially methylated in HD and annotated to miRNA that were differentially expressed in HD, were further investigated for an association with alcohol dependence in the validation cohort. We performed binomial logistic regression models of disease state (alcohol dependent or control) to CpG-site methylation levels and adjusting for the co-variates with significant between-group differences, i.e., gender and occurrence of any under treatment anxiety disorder. Cg01299774, the only investigated CpG-site, was significantly hypermethylated in alcohol dependence (p = 0.0261)(**Supplementary Table 6.**)(**Supplementary Figure 5.**).

## Discussion

In a DNA methylation association analysis in peripheral blood, we identify distinct CpG-sites associated with MIR708 and MIR4456 that are significantly differentially methylated in HD patients. Additionally, we demonstrate that hsa-miR-4456 associated methylation locus cg01299774 is differentially methylated in alcohol dependence, suggesting that it may be primarily associated with the addictive component observed in HD. We demonstrate that MIR4456 is considerably lower expressed in blood of HD patients using both univariate and multivariate analyses. Importantly, the differential expression status of MIR4456 is associated with epigenetic shifts in the identified methylation locus. Interestingly, this miRNA putatively targets several genes that are preferentially expressed in brain, amygdala and hippocampus and are involved in major neuronal molecular mechanisms thought to be relevant for HD, e.g., the oxytocin signalling pathway. Our findings suggest a role of MIR4456 in the pathogenesis of HD, which might be regulated by methylation shifts in the identified methylation locus.

To our knowledge, this is the first published study investigating associations between both DNA methylation and miRNA expression levels in HD. By choosing a methylome-wide study approach using the Illumina EPIC methylation array, it was possible to comprehensively investigate 8,852 miRNA-associated CpG-sites with regard to changes in methylation patterns in a hypothesis-free and unbiased manner. Specifically, MIR4456 had both a genome-wide significant methylation locus and a statistically significant abundance of nominally significant probes, providing evidence that MIR4456 associated methylation alterations occur in HD subjects. To our knowledge, no previous paper described the importance of MIR4456 in a context of psychopathologies. We identify that this miRNA is evolutionarily conserved with regard to primary sequence composition and predicted hairpin secondary structures from the advent of primates. In addition, we provide evidence that putative mRNA targets of MIR4456 are preferentially expressed in amygdala and hippocampus, two brain regions suggested by Kühn et al. to be implicated in the pathophysiology of HD [5].

The involvement of the oxytocin signalling pathway identified in this study appears to be significantly implicated in many of the characteristics defining HD as proposed by Kafka et al. [1], such as sexual desire dysregulation, compulsivity, impulsivity and (sexual) addiction. Mainly produced by the paraventricular nucleus of the hypothalamus and released by the posterior pituitary, oxytocin plays an important role in social bonding and sexual reproduction in both males and females [59]. Murphy et al. described elevated levels during sexual arousal [60]. Burri et al. found that intranasal oxytocin application in men resulted in an increase in epinephrine plasma levels during sexual activity and an altered perception of arousal [61]. Additionally, oxytocin has been proposed to inhibit the activity of the hypothalamic-pituitary-adrenal (HPA) axis during stress. Jurek et al. observed that oxytocin receptor-mediated intracellular mechanisms postpone the transcription of corticotropin-releasing factor (Crf) in the paraventricular nucleus, a gene strongly associated with the stress response [62]. Alterations in the oxytocin signalling pathway could explain findings by Chatzittofis et al., who observed HPA axis dysregulation in men with hypersexual disorder [3]. Furthermore, studies indicate that oxytocin may be involved in the pathophysiology of obsessive-compulsive disorder [63]. The interaction of oxytocin with the dopamine system, the HPA-axis and the immune system led to the postulation that individual differences in oxytocin levels are impacting addiction vulnerability [64]. While oxytocin has been previously associated with the regulation of social and aggressive behaviour, Johansson et al. further demonstrated that genetic variation in the oxytocin receptor gene (OXTR) impacted on the tendency to react to situations with elevated levels of anger under the influence of alcohol [65]. Lastly, Brüne et al. concluded that genetic variation in OXTR may contribute to explaining the pathophysiology of borderline personality disorder [66], a personality pathology characterized by severe impulsivity dysregulation [66].

MIR4456 may have an additional regulatory function in HD that was not revealed in the current study. In line with our findings, previous studies have reported associations of aberrant male sexual behaviour and genes involved in glutamatergic system in depressed individuals [67]. Furthermore, a potential role of the 3ʹ-5ʹ-cyclic adenosine mono phosphate (cAMP) levels in sexual receptivity was shown in female rats, by modulating the phosphoprotein-32 and leading to alterations of progestin receptors [68]. Interestingly, cAMP also regulates molecules associated with axon guidance [69], such as the B3gnt1 gene, which was associated with impaired sexual behaviour in male mice [70].

Our study has several limitations. First, while the Illumina EPIC methylation array has been proven to be highly reproducible and reliable [35], it would be valuable to confirm the differential methylation status of the MIR4456 associated CpG locus in an independent cohort of HD patients. Second, in the expression profiling of MIR4456 and MIR708, it was not possible to separate the plasma from the blood samples due to hemolysis of the erythrocytes. As such, the measured miRNA expression levels are based on miRNAs from both plasma and lysed blood cells. This, however, was the case for all our samples and should therefore not be a source of between-group bias. Third, putative mRNA targets of MIR4456 were identified by computer algorithms. Thus, further in vivo studies are needed to confirm the predicted MIR4456 targets to further assure the hypotheses drawn with regard to a MIR4456 associated regulation of oxytocin signalling. Fourth, while MIR4456 is shown to be differentially expressed in whole blood of HD patients, we do not know to what extent this finding reflects expression modifications occurring in brain. Fifth, we took the most important confounders available, such as depression, DST non-suppression status, CTQ total score and plasma levels of TNF-alpha, into consideration, in association analyses between methylation and hypersexuality. The patient population was as homogenous as found reasonable, excluding subjects with psychotic illness, alcohol or drug abuse, other psychiatric disease requiring immediate treatment and other severe physical illness. However, other potential confounding factors, e.g., dietary patterns, ethnicity or prandial states may be able to induce changes in methylation patterns as well [40]. Furthermore, as the mean difference in per cent DNA methylation between HD patients and healthy volunteers was only ~2.6% it can be called into question what impact such small methylation changes would have on physiology. There is, however, now a growing body of literature on specific genes, suggesting wide ranging transcriptional and translational consequences of subtle methylation changes (1–5%), especially in complex multifactorial conditions like depression or schizophrenia [71]. Lastly, due to improved annotation, our analysis was restricted to CpG sites on the Illumina 450K array. As such, this could be considered a limitation as we did not make use of the full potential of the EPIC array.

In conclusion, MIR4456 has significantly lower expression in HD. Our study provides evidence that DNA methylation at the cg01299774 locus is associated with the expression of MIR4456. This miRNA putatively targets genes preferentially expressed in brain tissue and involved in major neuronal molecular mechanisms thought to be relevant to the pathogenesis of HD. Our findings from the investigation of shifts in the epigenome contributes to further elucidating the biological mechanisms behind the pathophysiology of HD with special emphasis on MIR4456 and its role in oxytocin regulation. Further studies are needed to confirm and further elucidate the exact interplay between MIR4456 and the oxytocin signalling pathway in HD.
