# Supplementary material for: Hypermethylation-associated downregulation of microRNA-4456 in hypersexual disorder with putative influence on oxytocin signalling: A DNA methylation analysis of miRNA genes
Source: Epigenetics. 2019 Sep 22;15(1-2):145–60. doi: 10.1080/15592294.2019.1656157 (PMC6961682; doi:10.1080/15592294.2019.1656157)
Supplement: Supplemental Material [file kepi-15-1-2-1656157-s002.docx]

**Hypermethylation-associated downregulation of microRNA-4456 in hypersexual disorder with putative influence on oxytocin signaling: A DNA methylation analysis of miRNA genes**

Adrian E. Boström^1^, Andreas Chatzittofis^2^, Diana-Maria Ciuculete^1^, John N. Flanagan^4^, Regina Krattinger^5^, Marcus Bandstein^1^, Jessica Mwinyi^1^, Gerd A. Kullak-Ublick^5^, Katarina Görts Öberg^4^, Stefan Arver^4^, Helgi Schiöth^1^*, Jussi Jokinen^23^*

^1^Department of Neuroscience, Functional Pharmacology, Uppsala University, BMC, Box 593, 751 24, Uppsala, Sweden

^2^Department of Clinical Neuroscience/Psychiatry, Karolinska Institutet, Stockholm, Sweden

^3^Department of Clinical Sciences/Psychiatry, Umeå University, Umeå, Sweden

^4^Andrology/Sexual Medicine Group (ANOVA), Department of Medicine, Karolinska Institutet, Stockholm, Sweden

^5^Department of Clinical Pharmacology and Toxicology, University Hospital Zurich, University of Zurich, Switzerland

*Joint senior authors

University of Uppsala

Department of Neuroscience (Medical faculty)

Box 593, Husargatan 3, Uppsala, Sweden.

Email: adrian.bostrom@neuro.uu.se

Phone: +46 18 471 4136

Fax: +46 18 51 1540

**Supplementary Figure 1**. Prior-plots of ComBat adjustment


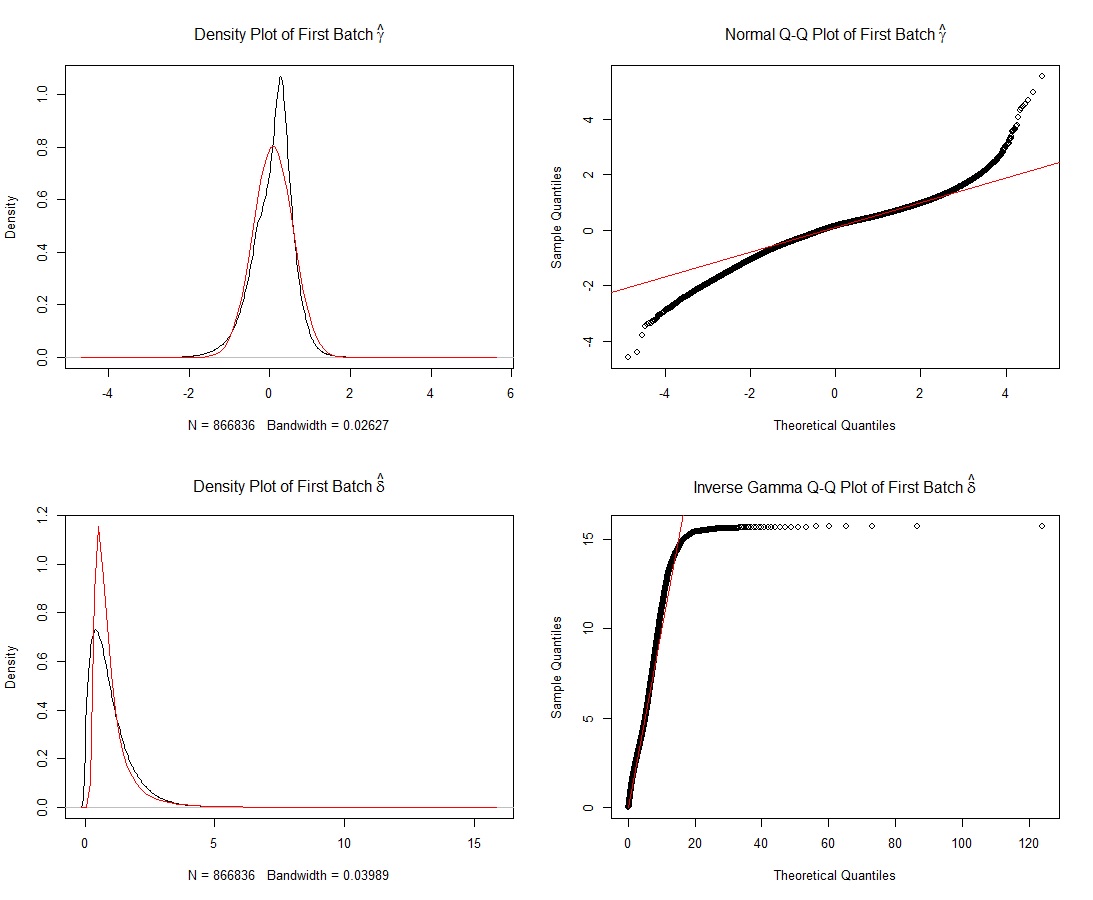


**Supplementary Figure 2**. PCA of methylation data after ComBat adjustment for Batch Effects

**
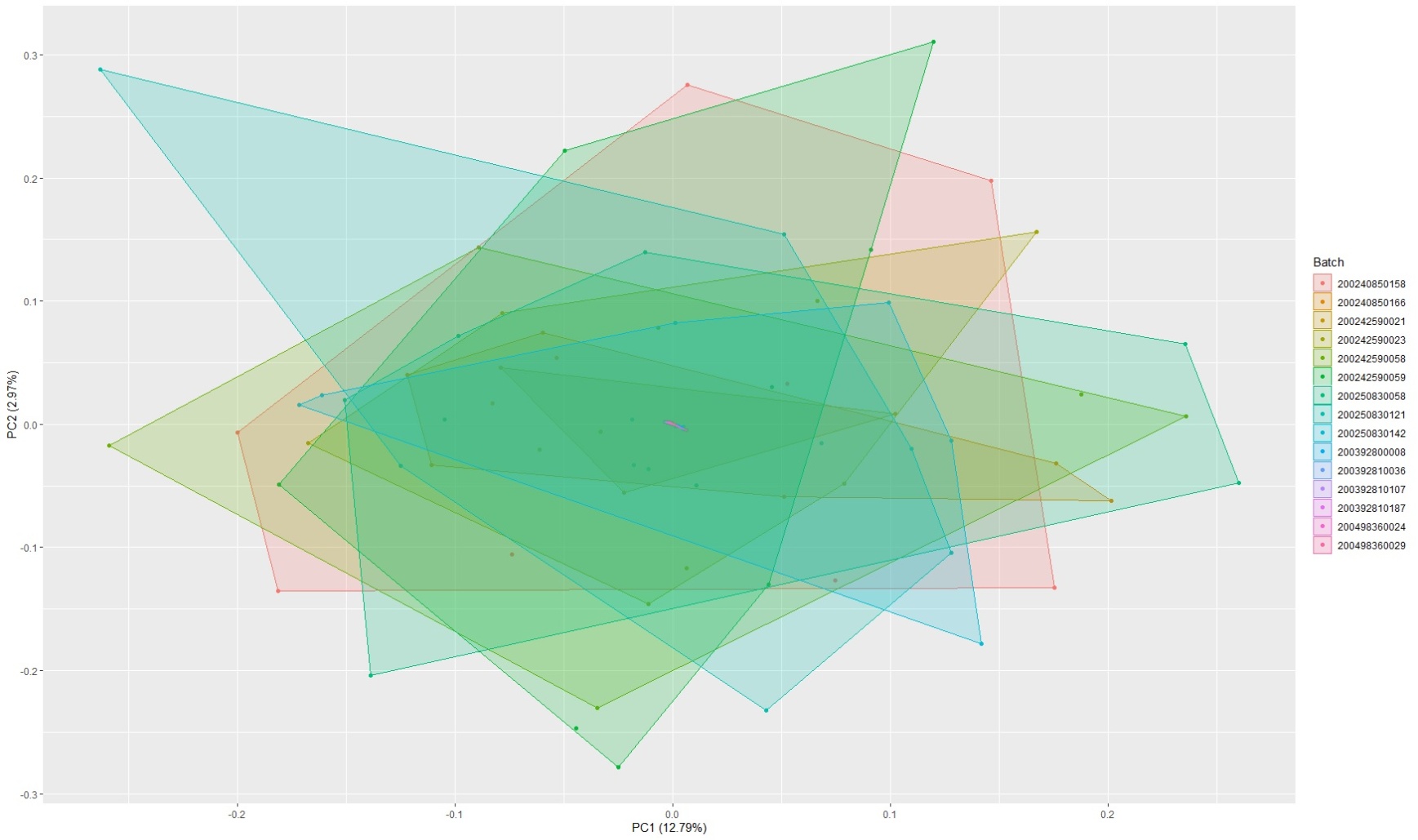
**

**Supplementary Figure 3**. Manhattan plot of epigenome-wide study


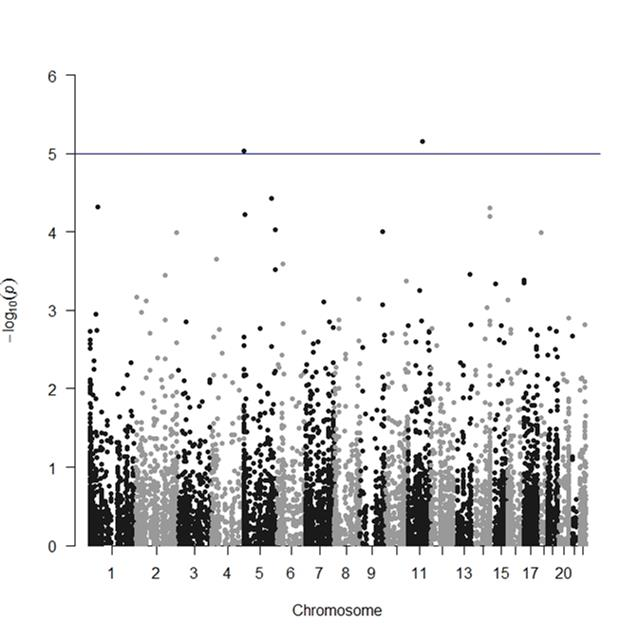


**Supplementary Figure 4**. Q-Q Plot of the moderated t statistic of the DNA methylation analysis of microRNA associated CpG-sites


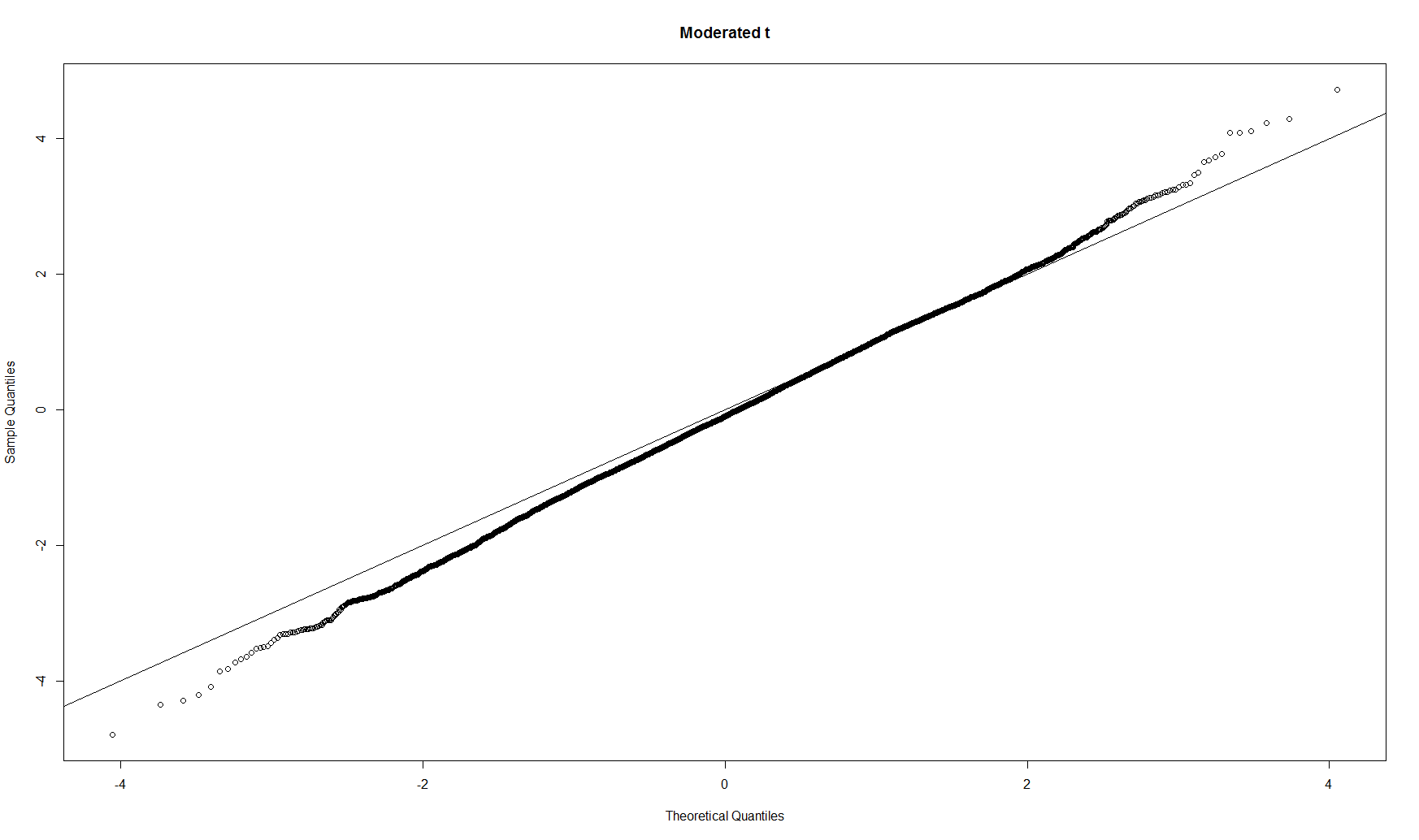


**Supplementary Table 1.** Clinical characteristics of subjects in the Validation cohort with alcohol dependence and controls

|  | Alcohol dependent | Controls | Statistics (t-test, Chisq.test) |
| --- | --- | --- | --- |
|  |  |  | , p value |
| N | 24 | 83 |  |
| Age (years) | 37.5 (12.6) | 41.2 (14.2) | *ns* |
| Men:Women, (n (%)) | 17 (70.8):7 (29.2) | 26 (31.3):57 (68.7) | **1.19E-03** |
| BMI, kg/m^2^ | 25.5 (3.0) | 25.4 (2.9) | *ns* |
| Depression (n (%)) | 6 (25.0) | 20 (24.1) | *ns* |
| Anxiety (n (%)) | 0 (0.0) | 8 (9.6) | **2.29E-02** |
| Bipolar disorder (n (%)) | 1 (4.2) | 5 (6.0) | *ns* |
| Post-traumatic stress disorder (n (%)) | 1 (4.2) | 6 (7.2) | *ns* |
| Values are shown as mean (SD) unless otherwise specified. P-values were calculated by means of unpaired t-tests or chi-squared tests, contrasting values for patients with alcohol dependence and controls. A one-tailed p-value <0.05 was considered significant. | | | |

**Supplementary Table 2.** Hypersexuality associated methylation changes in miRNA associated CpG-sites: Overrepresentation analysis

|  |  | **CpG-sites** | | | |
| --- | --- | --- | --- | --- | --- |
|  |  |  |  |  |  |
| **Gene** | **Transcript** | **Total** | **Sign.^a^** | ***p* (binomial)^b^** | ***p* (binomial, fdr.)^c^** |
| mir-133b | BX648566 | 6 | 4 | **8.64E-05** | 5.73E-02 |
| MIR4456 | NR_039661 | 33 | 8 | **1.76E-04** | 5.73E-02 |
| MIR124-1 | NR_029668 | 20 | 6 | **3.29E-04** | 7.85E-02 |
| MIR100HG | NR_024430 | 14 | 5 | **4.27E-04** | 7.85E-02 |
| MIRLET7BHG | BC036832 | 4 | 3 | **4.81E-04** | 7.85E-02 |
| MIR3943 | NR_037508 | 22 | 6 | **5.81E-04** | 8.13E-02 |
| MIR4277 | NR_036240 | 40 | 8 | **7.12E-04** | 8.71E-02 |
| MIR135A2 | NR_029678 | 5 | 3 | **1.16E-03** | 1.26E-01 |
| MIR4522 | NR_039748 | 18 | 5 | **1.55E-03** | 1.38E-01 |
| MIR1253 | NR_031654 | 18 | 5 | **1.55E-03** | 1.38E-01 |
| ^a^Number of nominally significantly hyper- or hypomethylated CpG sites per transcript (p<0.05) ^b^P-values for binomial tests, performed separately for each transcript, contrasting the total number of CpG-sites per transcript to the number of nominally significant CpG-sites for each transcript. ^c^P-values adjusted for multiple testing using the false-discovery rate (FDR)-method (979 transcripts in total) | | | | | |

**Supplementary Table 3.** Univariate analysis of differential expression in MIR708 and MIR4456 by hypersexuality disorder

|  | **Univariate analysis** | |
| --- | --- | --- |
|  | **(Kruskal-Wallis' test)** | |
| **miRNA** | ***chi-squared*** | ***p*** |
| MIR708^a^ | 2.6 | 1.10E-02 |
| **MIR4456^b^** | **13.9** | **1.94E-04** |
| Kruskal-Wallis' tests contrasting expression values of microRNA:s in patients with hypersexual disorder (HD) and healthy volunteers. ^a^45 HD were compared to 21 healthy volunteers ^b^55 HD were compared to 28 healthy volunteers Abbreviations: p, p-value | | |

**Supplementary Table 4.** Multivariate analysis by binomial logistic regressions of differential expression in MIR708 and MIR4456 in hypersexual disorder

|  | **MIR708** | | | |  | **MIR4456** | | | |
| --- | --- | --- | --- | --- | --- | --- | --- | --- | --- |
|  |  |  |  |  |  |  |  |  |  |
| **Parameter** | **coef.** | **Std. Error** | **z value** | ***p*** |  | **coef.** | **Std. Error** | **z value** | ***p*** |
| **Intercept** | -5.24 | 1.95 | -2.69 | 7.07E-03 |  | -2.46 | 1.89 | -1.30 | 1.92E-01 |
| **MIR708/MIR4456** | -2.15 | 1.19 | -1.81 | 7.08E-02 |  | **-2.52** | **1.04** | **-2.42** | **1.56E-02** |
| **Depression** | 17.00 | 2036.02 | 0.01 | 9.93E-01 |  | 17.77 | 1952.41 | 0.01 | 9.93E-01 |
| **DST non-suppression status** | 0.77 | 0.83 | 0.93 | 3.52E-01 |  | 1.28 | 0.81 | 1.58 | 1.14E-01 |
| **CTQ total** | 0.11 | 0.05 | 2.30 | 2.15E-02 |  | **0.06** | **0.03** | **1.80** | **7.22E-02** |
| **TNF-alpha (ng/L)** | 0.38 | 0.18 | 2.15 | 3.13E-02 |  | **0.33** | **0.19** | **1.74** | **8.14E-02** |
| Binomial logistic regressions contrasting microRNA expression levels by disease state (hypersexuality disorder or healthy volunteer), adjusting for both continuous variables, i.e. CTQ total and TNF-alpha (ng/L), as well as categorical co-variates, i.e. depression and DST non-suppression status. Abbreviations: coef., regression coefficient; p, p-value; Std. Error, standard error | | | | | | | | | |
|  |  |  |  |  |  |  |  |  |  |

**Supplementary Table 5.** Gene set overrepresentation analysis of MIR4456 putative binding targets – gene expression by tissue

| **Category** | **Term** | **Count** | **%** | **p.value** | **p.value (Benjamini.)** |
| --- | --- | --- | --- | --- | --- |
| **UP_TISSUE** | **Brain** | **569** | **51.3** | **9.4E-14** | **2.5E-11** |
| **UP_TISSUE** | **Amygdala** | **59** | **5.3** | **4.0E-5** | **5.4E-3** |
| **UP_TISSUE** | **Epithelium** | **201** | **18.1** | **4.4E-5** | **3.9E-3** |
| **UP_TISSUE** | **Teratocarcinoma** | **50** | **4.5** | **4.9E-4** | **3.2E-2** |
| **UP_TISSUE** | **Hippocampus** | **44** | **4.0** | **6.4E-4** | **3.4E-2** |
| UP_TISSUE | Spleen | 69 | 6.2 | 6.7E-3 | *ns* |
| UP_TISSUE | Cerebellum | 51 | 4.6 | 3.5E-2 | *ns* |
| UP_TISSUE | Human testis | 3 | 0.3 | 3.9E-2 | *ns* |
| UP_TISSUE | Spinal cord | 11 | 1.0 | 4.0E-2 | *ns* |
| UP_TISSUE | Endothelial cell | 8 | 0.7 | 4.5E-2 | *ns* |
| UP_TISSUE | Embryo | 28 | 2.5 | 4.6E-2 | *ns* |
| The online web tool 'ComIR' was used to computationally predict putative gene targets of MIR4456. Using the 'David Functional Annotation Bioinformatics MicroArray Analysis Tool', 1142 identified genes were investigated to see if there was a statistically significant abundance of genes expressed in different tissues. Abbreviations: Count, number of candidate genes expressed in a particular tissue; %, percentage of candidate genes expressed in a particular tissue; p.value, p-value; p.value (Bonf.), bonferroni adjusted p-value | | | | | |

**Supplementary Figure 5.** Heatmap of the number of overlapping MIR4456 associated genes in each pathway

**
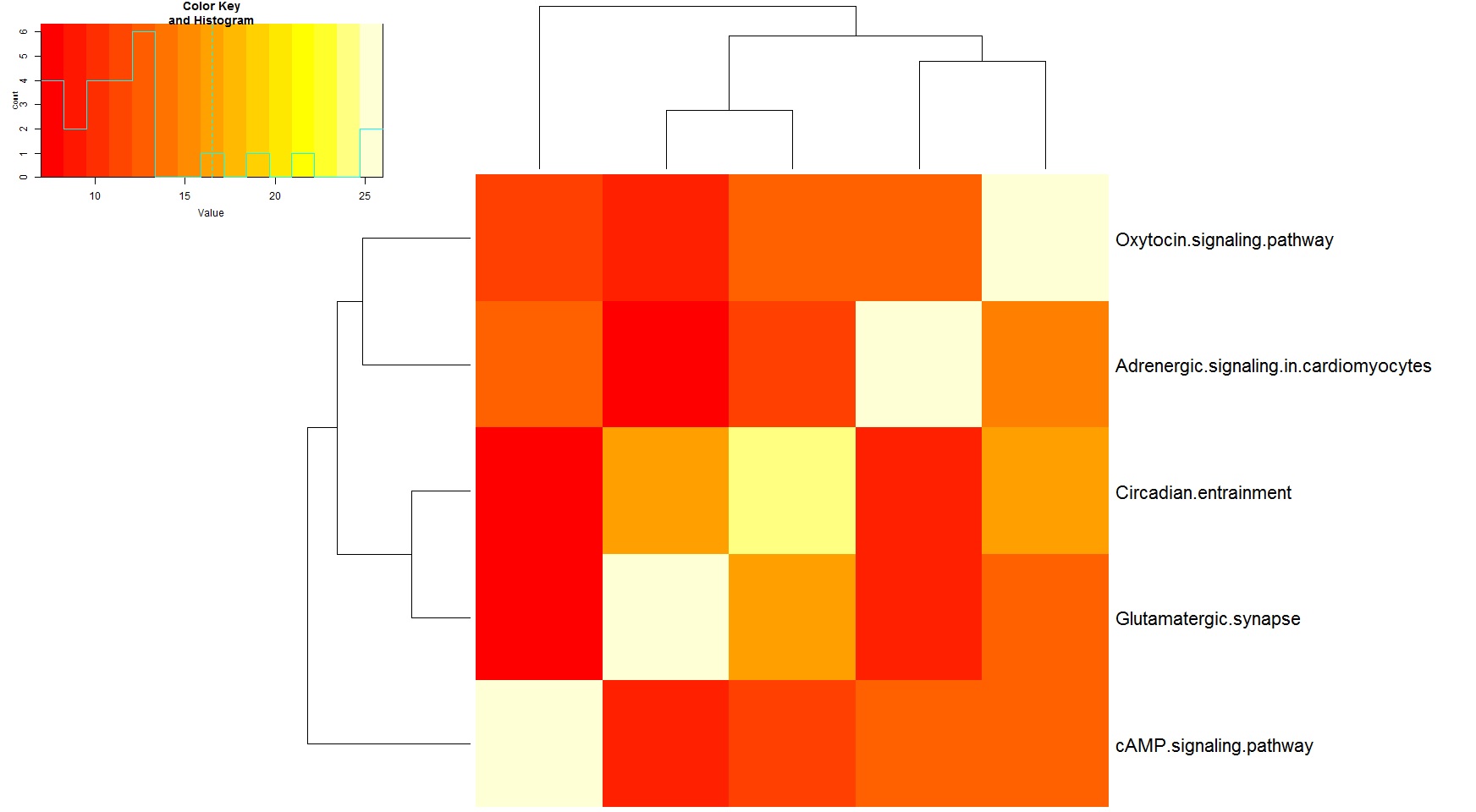
**

**Supplementary Figure 6**.

**
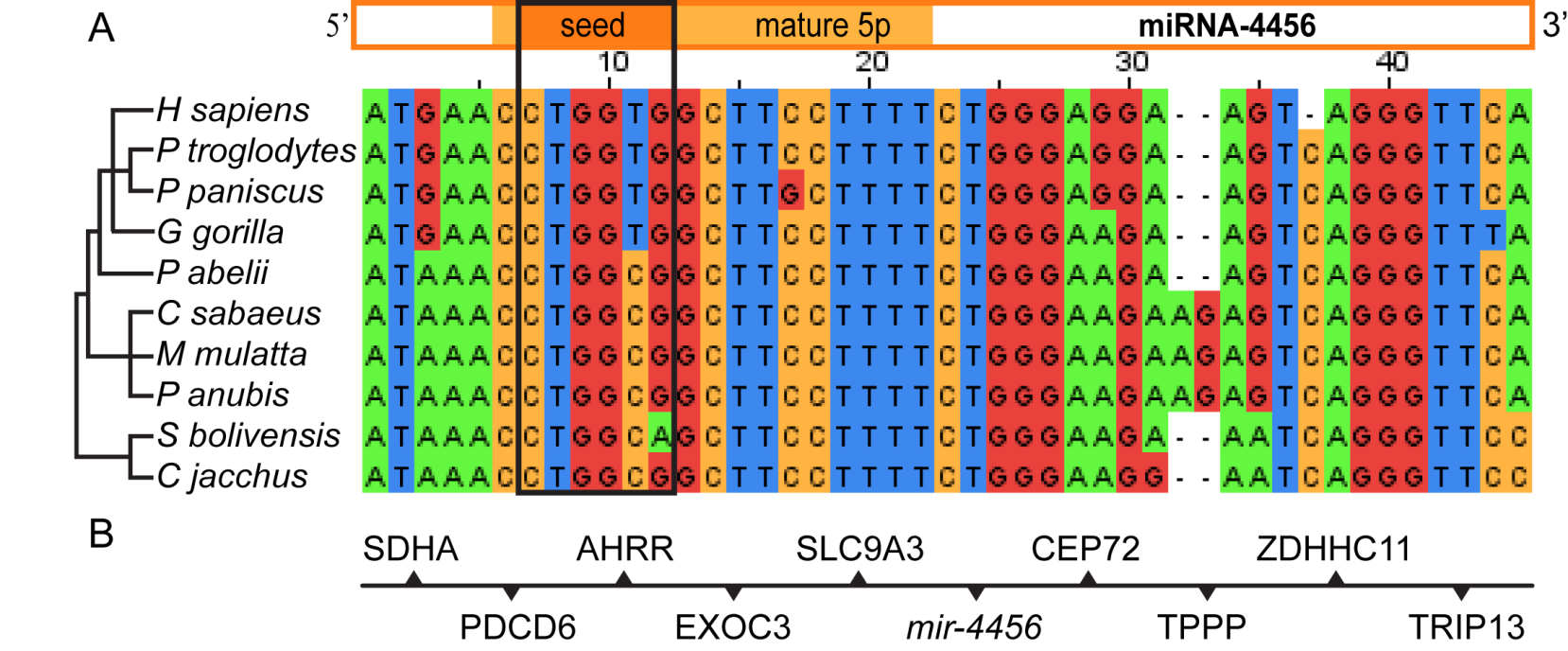
**

**Part A**. Conservation of genomic DNA of pre-miRNA-4456 with the mature 5p region and the seed positions (2-7) highlighted above the multiple sequence alignment. The cladogram represents the evolutionary relationship among the investigated organisms; the branch lengths are not relative to evolutionary distances. miRNA-4456 is identified solely in *Homo sapiens* in the miRBase sequence database. We investigated this genomic sequence in related species to assess if this is a conserved region. The miRNA4456 seed region is highly conserved within homininae and moderately conserved throughout Simiiformes. Additionally, the UNAFold Web Server predicts all of the sequence regions fold into hairpin turns. This may suggest that this region is conserved throughout primates. **Part B**. Gene synteny in the surrounding genomic region of miRNA-4456 in ten investigated species. The general gene synteny appears to be preserved throughout the species that have been mapped, however several of the species have incomplete mapping and gene annotation at this time.

**Supplementary Table 6.** Differential methylation analysis of cg01299774 in alcohol dependence compared to controls

| **Parameter** | **Coef.** | **Std. Error** | **z value** | **p.val** |
| --- | --- | --- | --- | --- |
|  |  |  |  |  |
| Intercept | -21.98 | 1711.57 | -0.01 | 9.90E-01 |
| cg01299774 | 6.06 | 2.72 | 2.23 | **2.61E-02** |
| Gender | 1.82 | 0.56 | 3.26 | **1.13E-03** |
| Anxiety Disorder (N) | 17.14 | 1711.57 | 0.01 | 9.92E-01 |
| Anxiety Disorder (Y) | -0.88 | 2683.45 | 0.00 | 1.00E+00 |
| Binomial logistic regression model of a binary outcome variable (alcohol dependence or control) to CpG site methylation and adjusting for gender and occurence of any anxiety disorder under treatment. Abbreviations: Coef., coefficient; p.val, p-value; Std. Error, standard error | | | | |

**Supplementary Figure 7.** Boxplot diagram of cg01299774 in alcohol dependence and controls

**
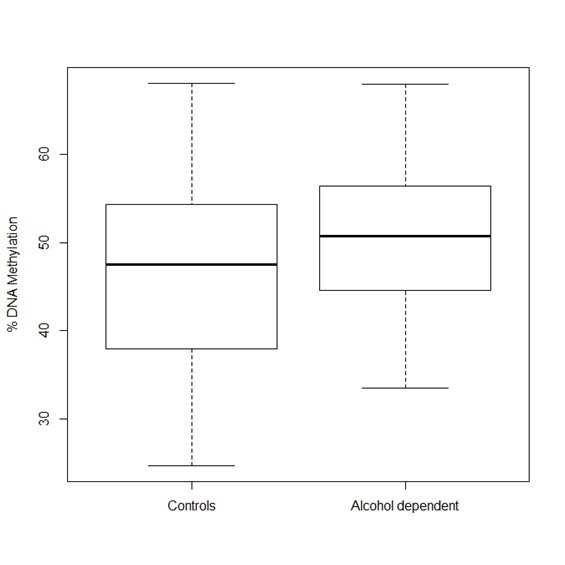
**

***Background correction, adjustment of type I and type II probes, removal of batch effects and probe exclusion***

Methylation idat files were first loaded into the R environment using the ‘read.metharray.exp’ function of the minfi package (v 1.18.2). Thereafter, the efficient NOOB method was selected to correct for background artifacts(1). Probes on the Illumina Methylation EPIC BeadChip array come in two different designs which differ in dynamic range and distribution of the DNA methylation pattern. We used the Beta Mixture Quantile Dilation (BMIQ) function of the wateRmelon package to adjust the methylation data for these probe type differences(2). In addition, the use of different analysis plates could result in undesired batch effects and we used the ‘ComBat’ function of the sva package to correct for this potential bias(3). Prior-plots and PCA analysis after ComBat adjustments are presented in Supplementary Figures 1-2. Moreover, methylation levels of CpG sites annotated to known SNP loci could be affected by single nucleotide polymorphisms (SNP:s)(4) and probes located on sex chromosomes have been shown to be more difficult to accurately normalize(5). 196,202 CpG sites were thus subsequently excluded as they were located on sex determining chromosomes or covering known SNP loci. In addition, 2,032 probes were also filtered out as 75% or more of the samples exhibited a detection p-value >10-5. After the probe exclusion steps outlined above, 668,602 CpG sites were included in the subsequent analysis.

***Correction for white blood cell type heterogeneity***

DNA methylation measured in whole blood is composed of different cell populations(6). Rask-Andersen et al. showed that changes in leukocyte fractions could introduce significant variability in the DNA methylation pattern, an effect that could bias downstream analyses. It is thus important to adjust the global DNA methylation pattern for white blood cell type heterogeneity(7). For this purpose, we used the ‘champ.refbase’ function of the ChAMP package, which implements a statistical procedure of the Houseman algorithm to estimate the relative proportions of CD4+ and CD8+ T cells, monocytes, granulocytes, B cells and natural killer cells based on the DNA methylation pattern(8). Using the estimated relative proportions of leukocyte subpopulations, methylation data was thereafter adjusted for white blood cell type heterogeneity in a method similar to regression calibration (‘RefBaseEWAS’).

***Criteria of sample exclusion***

To investigate the global DNA methylation pattern for sample outliers, the ‘PCA’ function of the FactoMineR package was used(9). 7,547 probes were further studied and included in the covariance matrix based on a threshold of 0.2 and a 95% reference range, as performed by Voisin et al(10). The first principal component explained 20,4% of the total variance and successively studied vectors did not add significantly to the total variance. Outliers were identified by visual inspection of the graphical display of the first principal component, resulting in seventeen samples being excluded from further analysis.

***References***

1. Triche TJ, Weisenberger DJ, Van Den Berg D, Laird PW, Siegmund KD. Low-level processing of Illumina Infinium DNA Methylation BeadArrays. Nucleic Acids Res. 2013;41(7).

2. Teschendorff AE, Marabita F, Lechner M, Bartlett T, Tegner J, Gomez-Cabrero D, et al. A beta-mixture quantile normalization method for correcting probe design bias in Illumina Infinium 450 k DNA methylation data. Bioinformatics. 2013;29(2):189–96.

3. Johnson WE, Li C, Rabinovic A. Adjusting batch effects in microarray expression data using empirical Bayes methods. Biostatistics [Internet]. 2007;8(1):118–27. Available from: http://www.ncbi.nlm.nih.gov/pubmed/16632515

4. Chen YA, Lemire M, Choufani S, Butcher DT, Grafodatskaya D, Zanke BW, et al. Discovery of cross-reactive probes and polymorphic CpGs in the Illumina Infinium HumanMethylation450 microarray. Epigenetics. 2013;8(2):203–9.

5. Fortin J-P, Labbe A, Lemire M, Zanke BW, Hudson TJ, Fertig EJ, et al. Functional normalization of 450k methylation array data improves replication in large cancer studies. Genome Biol [Internet]. 2014;15(12):503. Available from: http://genomebiology.com/2014/15/11/503

6. Reinius LE, Acevedo N, Joerink M, Pershagen G, Dahlen SE, Greco D, et al. Differential DNA methylation in purified human blood cells: implications for cell lineage and studies on disease susceptibility. PLoS One [Internet]. 2012;7(7):e41361. Available from: http://www.ncbi.nlm.nih.gov/pubmed/22848472%5Cnpapers3://publication/doi/10.1371/journal.pone.0041361

7. Rask-Andersen M, Bringeland N, Nilsson EK, Bandstein M, Búcaro MO, Vogel H, et al. Postprandial alterations in whole-blood DNA methylation are mediated by changes in white blood cell composition. Am J Clin Nutr. 2016;104(2):518–25.

8. Houseman EA, Accomando WP, Koestler DC, Christensen BC, Marsit CJ, Nelson HH, et al. DNA methylation arrays as surrogate measures of cell mixture distribution. BMC Bioinformatics [Internet]. 2012;13(1):86. Available from: http://www.biomedcentral.com/1471-2105/13/86

9. Lê S, Josse J, Mazet F. Package ‘ FactoMineR .’ J Stat Softw [Internet]. 2008;25(1):1–18. Available from: http://www.jstatsoft.org/v25/i01/

10. Voisin S, Almén MS, Zheleznyakova GY, Lundberg L, Zarei S, Castillo S, et al. Many obesity-associated SNPs strongly associate with DNA methylation changes at proximal promoters and enhancers. Genome Med [Internet]. 2015;7(1):103. Available from: http://genomemedicine.com/content/7/1/103%5Cnhttp://www.ncbi.nlm.nih.gov/pubmed/26449484%5Cnhttp://www.pubmedcentral.nih.gov/articlerender.fcgi?artid=PMC4599317
